# Identifying lncRNA-mediated regulatory modules via ChIA-PET network analysis

**DOI:** 10.1186/s12859-019-2900-8

**Published:** 2019-05-29

**Authors:** Denise Thiel, Nataša Djurdjevac Conrad, Evgenia Ntini, Ria X. Peschutter, Heike Siebert, Annalisa Marsico

**Affiliations:** 1 Max Planck Institute for Molecular Genetics, Berlin, Ihnestraße 63-73, Berlin, 14195 Germany; 2 Department of Mathematics and Informatics, Freie Universität, Berlin, Arnimallee 7, Berlin, 14195 Germany; 3 Zuse Institute Berlin (ZIB), Takustraße 7, Berlin, 14195 Germany; 4 Institute of Computational Biology (ICB), Helmholtz Zentrum München, Ingolstädter Landstraße 1, Oberschleißheim, 85764 Germany

**Keywords:** lncRNA, Modules, Network analysis, ChIA-PET, Gene regulation

## Abstract

**Background:**

Although several studies have provided insights into the role of long non-coding RNAs (lncRNAs), the majority of them have unknown function. Recent evidence has shown the importance of both lncRNAs and chromatin interactions in transcriptional regulation. Although network-based methods, mainly exploiting gene-lncRNA co-expression, have been applied to characterize lncRNA of unknown function by means of ’guilt-by-association’, no strategy exists so far which identifies mRNA-lncRNA functional modules based on the 3D chromatin interaction graph.

**Results:**

To better understand the function of chromatin interactions in the context of lncRNA-mediated gene regulation, we have developed a multi-step graph analysis approach to examine the RNA polymerase II ChIA-PET chromatin interaction network in the K562 human cell line. We have annotated the network with gene and lncRNA coordinates, and chromatin states from the ENCODE project. We used centrality measures, as well as an adaptation of our previously developed Markov State Models (MSM) clustering method, to gain a better understanding of lncRNAs in transcriptional regulation. The novelty of our approach resides in the detection of fuzzy regulatory modules based on network properties and their optimization based on co-expression analysis between genes and gene-lncRNA pairs. This results in our method returning more *bona fide* regulatory modules than other state-of-the art approaches for clustering on graphs.

**Conclusions:**

Interestingly, we find that lncRNA network hubs tend to be significantly enriched in evolutionary conserved lncRNAs and enhancer-like functions. We validated regulatory functions for well known lncRNAs, such as MALAT1 and the enhancer-like lncRNA FALEC. In addition, by investigating the modular structure of bigger components we mine putative regulatory functions for uncharacterized lncRNAs.

**Electronic supplementary material:**

The online version of this article (10.1186/s12859-019-2900-8) contains supplementary material, which is available to authorized users.

## Introduction

Long non-coding RNAs (lncRNAs), an heterogeneous group of non-coding transcripts longer than 200 nucleotides, are expressed in a time- and tissue-specific fashion and have been shown to regulate cellular processes such as development, imprinting, X-chromosome inactivation, cancer and immunity [1, 2]. The discovery of extensive transcription of these non-coding transcripts provides an important new perspective on the centrality of RNAs in gene regulation [3]. To date, next-generation sequencing data generated by several consortia, such as the ENCODE [4] or FANTOM5 [3] leads to an estimate of the number of potential lncRNA transcripts of about 20000. Although only a smaller fraction of such transcripts might be functional, and despite the substantial progress in mapping lncRNAs, the detailed functional mechanisms for most of them remain elusive [2]. The gap in the understanding of the functional roles of the lncRNAs has largely been due to their poor evolutionary conservation, but also to the limited ability of tools to characterize lncRNA interactions with either proteins, DNA and RNA on a large scale. Concomitant with the increasing number of lncRNAs, a number of resources collecting and curating functional information about lncRNAs have been built in recent years [5–8].

It has been shown, among others, that lncRNAs can regulate the expression either of their neighboring genes in *cis*, or of more distant genes in *trans*. LncRNAs may function via binding to RNA Binding Proteins (RBPs), such as chromatin regulators that can bind both RNA and DNA, or by interactions with other nucleic acids [9].

A major category of well-studied functional lncRNAs is those implicated in coordinated gene silencing, either in *cis* (e.g. the lncRNA Xist, involved in X-chromosome inactivation) or in *trans* (e.g. HOTAIR). Both XIST and HOTAIR have been shown to mediate epigenetic mechanisms of gene silencing [10, 11].

Genome-scale mapping of histone modifications and enhancer-binding proteins has helped to identify lncRNAs involved in gene activation. Enhancers are regulatory sequences that can activate gene expression, and their function depends on the interplay between DNA sequences, DNA-binding proteins, and architecture [12]. In the last five years, the functional landscape of enhancers has become more complex with the evidence that active enhancers can transcribe structured lncRNAs. A recent study performed loss-of-function experiments and found 7 of 12 enhancer-transcribed lncRNAs affecting expression of their cognate neighboring genes [13]. More recently, HOTTIP, an enhancer-like lncRNA, has been discovered to directly interact and activate the WDR5 protein [10], a key component of the mixed lineage leukemia-Trx complex. In other cases lncRNAs activate a neighboring lncRNA, e.g., JPX regulates transcriptional activation of XIST on chromosome X [10]. Long noncoding RNAs with activating function may recruit transcriptional activators involved in the establishment of chromosome looping between the lncRNA loci and regulated promoters, such as the mediator complex [14].

The architectural landscape of the nucleus has a profound influence on gene regulation. Chromosome conformation capture technologies, such as 3C, Hi-C, 4C, Capture-C and Chromatin Interaction Analysis by Paired-End Tag Sequencing (ChIA-PET) have revealed elements that are distally located either on the same or separate chromosomes, to be proximal in the three dimensional nucleus [15]. The effect of such contacts, especially when they correspond to enhancer-promoter or promoter-promoter interactions, mediated by PolII or other factors, is an area of intense research [15]. There is evidence that enhancer-promoter interactions might be induced by chromatin looping and mediated by enhancer-like non-coding RNAs (ncRNAs), and that the ChIA-PET technique is suitable to detect them [10, 16].

Additional evidence on potential functions of lncRNAs have been obtained from methodologies which rely on expression patterns and “Guilt by Association”: transcripts sharing common expression patterns are expected to be co-regulated or share common pathways [17, 18]. Most of these methods build a coding-non-coding co-expression network, in which a node represents a molecule and an edge an expression correlation. Such a network is used to identify cellular modules involving both protein coding genes and lncRNAs, and the unknown function of lncRNAs is predicted by transferring functional annotation (e.g. Gene Ontology (GO) terms) from protein coding genes [10, 17, 19]. These approaches however detect statistical associations, and thus do not directly contribute to an understanding of detailed mechanisms of lncRNA-mediated gene regulation.

In this study we focused on lncRNA regulatory functions in the cell nucleus and constructed the chromatin interaction network involving lncRNAs, genes and other genomic regions using ChIA-PET data in the K562 cell line, which compared to HiC has higher genomic resolution. ChIA-PET combines ChIP with chromatin capture technology to detect interactions between genomic regions mediated by a transcription factor of interest [20]. Here, we focus on the Polymerase II (Pol II)-mediated chromatin network, as it is directly linked to transcriptional regulation. A natural representation of these data amenable to efficient analysis are complex networks, where nodes represent DNA segments or Paired-End Tags (PETs), and edges represent ChIA-PET interactions between two PETs. The analysis of chromatin interaction networks has been an area of active research in the last years, but very few studies have employed network analysis and clustering methods to study chromatin interaction networks [15, 21].

For many biological networks, including gene regulatory networks, the evaluation of well-established node characteristics, in particular centrality measures, are highly suitable for identification of functionally essential elements [22]. Similarly, modular organization is believed to be a generic property of such networks, allowing to uncover subnetworks responsible for a specific function. In gene regulatory networks for instance, modules often correspond to groups of interconnected cis-regulatory elements.

**Fig. 1:**
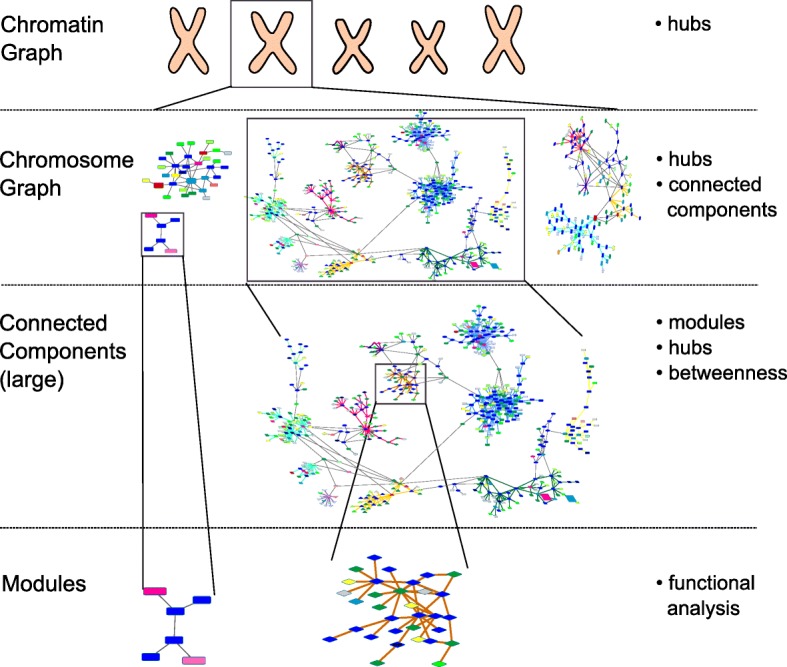
Overview of the hierarchical graph analysis. The different levels represent a zoom into more detail in the graph, starting with the chromatin graph at the top, then focusing on a single chromosome followed by large connected components and lastly modules detected in the large component using the MSM algorithm or as small connected components of the corresponding chromosome. On the right, we list the different analysis steps performed at each level, focusing only on degree centrality on the level of the chromatin graph, then adding in consideration of connectivity properties as well as module detection and finally considering molecular information to assess possible functional interactions within modules. Node shapes are arbitrary and node colors symbolize different node annotations

We developed a hierarchical network analysis approach to compute centrality properties of lncRNAs in the chromatin network, followed by a focus on the connected components of the chromosome graphs and finally reaching the level of density-based modules, that are amenable to a detailed analysis in their entirety (Fig. 1). Specifically, to identify these potential lncRNA-mediated functional modules, we implement a modified version of our previously developed Markov State Models (MSM) clustering approach [23, 24], which aims at identifying subgraphs of high connectivity. Compared to previous methods we do not rely on lncRNA-mRNA co-expression for network building, neither for clustering, but only on the topology and properties of the chromatin graph. Co-expression information is incorporated only in a second step by the algorithm to fine-tune the final network partition, based on the expectation that genes and lncRNAs which are spatially coordinated and contained in the same functional module also have related expression patterns. To our knowledge, this is the first approach that defines modularity in a mRNA-lncRNA interaction network based on chromatin interactions and uses the added value of co-expression to refine interacting modules and characterize unknown regulatory RNAs.

We compare our method with other state-of-the-art graph clustering methods, and show that MSM clustering is superior in returning clusters corresponding to genuine regulatory modules, i.e. whose members exhibit a high correlation in expression between gene-gene, lncRNA-gene and lncRNA-lncRNA node pairs. We evaluated our approach by matching modules and interactions to lncRNAs of known function, such as ncRNA-a3, FALEC, Xist and MALAT1 [9]. LncRNAs transcribed from enhancer regions exhibit either a high degree or high betweenness centrality, highlighting their regulatory potential in the leukemia-specific network. Finally, we inspect potential functions of lncRNA modules in big chromosome connected components, making our strategy a valuable tool towards functional annotation of lncRNAs with functions in transcriptional gene regulation.

## Methods

### Data collection and Pre-processing

**ChIA-PET Data.** The Pol II ChIA-PET interaction network in the K562 cell line was build based on the already processed interaction files downloaded from the ENCODE project website. Interacting pairs of genomic regions from this files corresponds to two nodes linked by an edge in our network. The data corresponding to two different ChIA-PET replicates were downloaded and only interactions supported by both replicates were retained for further analysis.

**Fig. 2:**
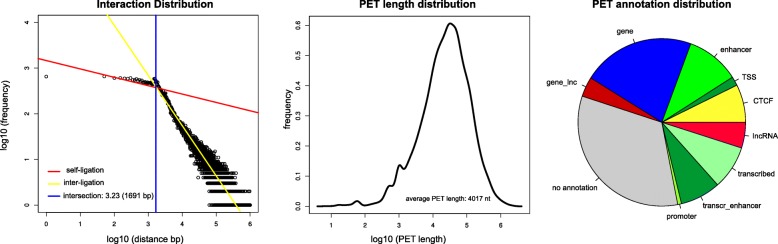
Filtering of interacting regions. Left panel: Fitted mixture model to classify PETS in self-ligation and inter-ligation. Middle panel: Distribution of inter-ligation PET fragments’ length. Right panel: Relative abundance of ChIA-PET fragments across different genomic annotations on the chromatin network

**Filtering of PET interactions.** As we were interested in *cis* long-range interactions we filtered out the 1.8*%* inter-chromosomal PET interactions before further analysis. Also we excluded the so-called self-ligation PETs from further analysis [25], as they represent an artifact of ChIA-PET experiments, and originate from self-circularization ligation of the same chromatin fragment resulting in ChIA-PET sequences with both tags mapped within a short genomic distance of each other. In order to distinguish between self-ligation PETs and inter-ligations PETs, which actually correspond to two distinct interacting chromosomal regions, we performed a similar analysis to Li et al. [25]. We computed the genomic distances between PETs and plotted their frequency in each genomic bin on a log-log scale. The intersection of two fitted lines at 1691 nt was taken as distance cutoff to distinguish self-ligation from inter-ligation PETs, which seem to follow two distinct power-law distributions (Fig. 2 left). Self-ligation interactions, with distances below this cutoff, were discarded from further analysis.

**Expression analysis of lncRNAs and genes.** Expression levels of both lncRNAs and protein-coding genes in K562 were computed from the corresponding alignment file of RNA sequencing (RNA-seq) from the Cold Spring Harbor Lab (CSHL) ENCODE track (chromatin fraction). Genomic annotation of lncRNAs and genes was taken from Gencode v24. Coordinates were lifted over the hg19 human genome assembly as all other annotations were on hg19. Read counts in protein-coding genes and lncRNAs were obtained by means of htseq-count [26] for two different replicates with default parameters (stranded, skip all reads with alignment quality lower 10, overlapping reads handled as union), using only complete gene regions (introns included) from the annotation file and converted to Reads Per Kilobase of transcript, per Million mapped reads (RPKMs). Only genes with an RPKM >0.041 and lncRNAs with RPKM >0 in both replicates or RPKM >0.041 in at least one replicate were considered ’detected’ and retained for further analysis. The 0.041 threshold was determined by looking at the bimodal distribution of the log RPKM expression values of all genes and corresponds to the local minimum separating the two modes.

**Fig. 3:**
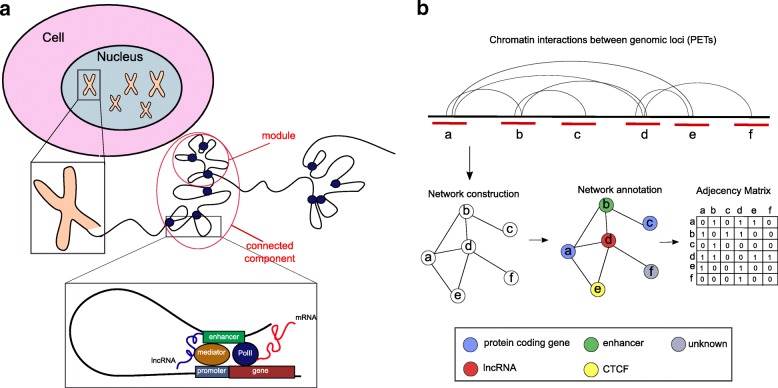
Construction and annotation of the chromatin graph. **a** Modular organization of chromatin on each chromosome with highlight on looping between regulatory elements such as enhancers and promoters mediated by PolII, Mediator and nascent lncRNAs. **b** Steps involved in network construction and annotation from ChIA-PET data

**Network construction and annotation.** PETs representing interacting genomic regions were annotated as ’gene’, and assigned their corresponding official gene symbol if they overlapped the genomic coordinates of annotated protein-coding genes from Gencode. PETs were annotated as ’lncRNA’ if they overlapped the genomic coordinates of annotated lncRNAs from Gencode. Given that the resolution of the ChIA-PET data is in the order of few kilobases, it could occur that interacting PETs might cover wide genomic regions with more than one annotated gene/lncRNA. In addition, ChIA-PET data are not strand-specific, therefore they might overlap with two or more genes/lncRNAs located on different strands. PETs corresponding to more than one gene/lncRNA location, either on the same or the opposite strand, were annotated with both gene and lncRNA names. Chromatin states in K562 from the *chromHMM* software genome segmentation [27] downloaded from the ENCODE website were also used to annotate interacting PETs in the network as ’enhancer’, ’weak enhancer’, Transcription Start Site (’TSS’), ’promoter flanking’, ’CTCF’, ’transcribed’ and ’repressed’ (Fig. 3b). The assignment ’repressed’ was ignored because in a network containing interactions mediated by Pol II, repressed regions hold no information. It could occur that the same PET overlapped with many different features. In this case annotations were merged. For example a PET overlapping both an annotated lncRNA and an enhancer region was defined as ’lncRNA_enhancer’. If PETs did not overlap with any annotated gene, lncRNA or chromatin state, were labeled as *unknown*. Annotated PETs were represented as nodes in the network and an interaction between PETs as an edge. A global (0,1)-adjacency matrix was build to describe the overall graph, called from now on *chromatin graph*. The number of rows and columns of the adjacency matrix represents the number of genomic regions involved in at least one ChIA-PET interaction. A 0-entry in the matrix cell corresponds to no interactions between any two PETs overlapping with these regions, while a 1-entry corresponds to a ChIA-PET interaction. A schematic view of the steps described above is given in Fig. 3b.

For gene disease annotation the disease databases OMIM [28] and DisGenet [29] were used. Disease annotation data for lncRNAs was taken from the database lncRNADisease (as of June 2015) [30], where we used both experimentally validated associations between lncRNAs and diseases, as well as predicted associations. LncRNAs that were part of positionally conserved pairs of genes and lncRNAs were obtained from [31]. Additional annotations, such as functional lncRNAs in K562, VISTA and FANTOM5 enhancers, enhancers annotated from other sources [32], cancer risk Single Nucleotide Polymorphism (SNP) annotation and mouse orthologs we taken form Liu et al. [33].

### Network analysis of the chromatin graph

**Centrality measures** For graph analysis we use standard graph concepts of interest for biological network analysis, see, e.g., [34] and [22]. To identify nodes of potential functional importance, we first look for nodes with a high degree, i.e., with a high number of incident edges, also called hubs. For each node *v* in a graph *G*=(*V*,*E*) we calculate the number *d*(*v*) of edges incident to *v* and call it its degree or degree centrality. For capturing the importance of a node *v*∈*V* as an efficient connector between other nodes in the network we consider its betweenness centrality. It is defined as $b(v)= \sum _{s\neq v\neq t}(\sigma _{st}(v)/\sigma _{st})$, where *σ*_*st*_ is the number of shortest paths from node *s* to node *t* and *σ*_*st*_(*v*) is the number of such paths that pass through *v*.

**MSM clustering for module detection** Apart from single node characteristics, we are interested in sets of nodes forming functional units. A connected component *C*=(*V*_*C*_,*E*_*C*_) of a graph is defined as an inclusion-wise maximal subgraph of *G* such that there exists a path between *v* and *w* for all vertices *v*,*w*∈*V*_*C*_. If such a component is rather large, it often consists of so-called modules, i.e., subgraphs that have a high intra-connectivity but are only sparsely connected to the rest of the network. The modules are thus good candidates for functional units.

In this paper, we apply the MSM clustering method developed in [23, 24] on large connected components for finding modules. It is based on finding markov state models of a time-continuous random walk process. More precisely, it identifies modules as regions of the network where the process is metastable, i.e. trapped for a longer period of time. To this end, the number of network modules can be induced from the number of dominant eigenvalues of the generator matrix that governs the dynamics of the random walk process. Unlike most of the common approaches, MSM finds fuzzy instead of complete partitions of the network into modules, where some nodes are not uniquely assigned to exactly one of the modules, but can belong to several modules or to none. This allows to also capture intermodular nodes whose functional significance lies in mediating interactions between modules.

For every node *x* we can calculate a value *q*_*i*_(*x*) as the random walk based probability of affiliation of a node *x* to a module *M*_*i*_. We then use a free parameter *θ* to refine the partitioning, i.e. we assign a node *x* to a module *M*_*i*_ if *q*_*i*_(*x*)≥*θ*. If *θ*=1 we obtain subgraphs exhibiting the strongest cohesiveness. By decreasing *θ* we expand modules until we reach a full partitioning of a graph by associating each vertex from the transition region with exactly one module it most likely belongs to. Fuzzy affiliation functions *q*_*i*_,*i*=1,…,*m* can be obtained by solving sparse, symmetric and positive definite linear systems ([23, 35]).

Another free parameter is a resolution parameter *α*, indicating how densely connected the modules we are interested in finding should be. For high values of *α* the method finds dominant, highly intraconnected modules and by decreasing *α* it finds also less pronounced modules. This is connected to the timescale at which the random walk leaves the transition region. It can be originally set according to the gap in the dominant spectrum of the generator of the random walk and then varied to observe the effect on the modules. In our application, it usually ranges from 100 to 2000.

**Empirical Optimization criteria** The parameters *θ* and *α* allow for an adaptation of the clustering to the specific application by integrating additional information on the networks nodes beyond the characteristics given by the network topology. Since we are looking for regulatory units involving lncRNAs, we chose to compare co-expression levels of intra- versus inter-modular gene-gene, lncRNA-gene and lncRNA-lncRNA pairs in order to find the best clustering parametrization. We argue that elements within the same module should have more correlated expression profiles, indicating co-regulation or potential mutual regulation, whereas intermodular node pairs are more independently regulated. In detail, we performed the MSM clustering for connected components from all chromosome graphs for a range of *α* and *θ* combinations. We chose the best combination by optimizing an empirical objective function (Eq.1) defined by the ratio of the median intra-module Mutual Information (MI) and the inter-module MI for all gene pairs in the connected component.

1 $$  \{\theta, \alpha\}_{best}={argmax}_{\substack{\theta,\alpha}} \frac{median(intra\_MIs)}{median(inter\_MIs)}  $$

MI values between variables *X*, RPKM expression vector of gene1/lncRNA1 across 24 tissues and *Y*, RPKM expression vector of gene2/lncRNA2 across 24 tissues, is defined in terms of their marginal Shannon entropies *H*(*X*) and *H*(*Y*) and their joint entropy *H*(*X*,*Y*), as implemented in *scikit-learn* python package:

2 $$  MI(X,Y)=H(X)+H(Y)-H(X,Y)  $$

The entropy can explicitely be written as:

3 $$  H(X)=-\sum_{i=1}^{n} p(x_{i})lnp(x_{i})  $$

where *x*_*i*_ are the possible values of random variable *X* with probability mass function *p*(*X*). In detail, we apply a Gaussian smoothing to the histogram from the distributions of *X*, *Y* and *joint*(*X*,*Y*) and compute the entropy rather on the continuous distribution as described in [36].

LncRNAs tended to be more cell type-specific than protein-coding genes (Additional file 1: Figure S1a, b) and this might bias the MI computation (Additional file 1: Figure S1c). Computing the MI ratio on all gene pairs provides a more robust value. The reported ratio in Eq.1 for a connected component serves also as indicator for the quality of the clustering, where a high score implies a better partitioning with respect to MI and a ratio of at least one is expected for biologically meaningful clusterings. The best values for *α* and *θ* for each inspected connected component are reported in the table of Additional file 2, together with other properties of the detected clusters. We observe that generally clusterings with *θ*=0.7 and small *α* (around 100–500), allowing more sparsely connected and relaxed modules, provide the highest MI ratio.

### Comparison with other clustering methods

We compared our MSM clustering approach to other state-of-the-art clustering methods with respect to the mutual information ratio, which reflects our expectation that nodes connected in a module have correlated expression profiles. It is important to note again that our primary goal is to find modules that could represent functional units. To allow for and strengthen such an interpretation we consider co-expression of the involved nodes. The MSM approach allows us to integrate this aspect directly in the module detection by optimizing its parameters using MI ratios. This is a distinct advantage of our chosen method that is not directly reproducible by most commonly used clustering methods. We nevertheless need to consider whether other approaches might still yield more appropriate modules with respect to their co-expression in order to choose the most suitable method for our analysis.

We used the following methods and their implementation from the R *igraph* package [37]:

- *cluster_fast_greedy* function (FG), which finds dense subgraphs by directly optimizing a modularity score *Q*. Given a set of modules, *Q* is computed as the ratio between the fraction of within-community edges versus the expected fraction of connections for the randomized network [38].
- clustering via Edge Betweenness (EB), *cluster_edge_betweenness* function, which is based on iteratively removing edges with highest edge betweenness from the graph [39], in order to hierarchically split the graph into modules.
- leading eigenvalue clustering algorithm (EV), *cluster_leading_eigen* function, which implements the popular graph clustering method from Newman [40]. This method finds network modules by calculating the leading non-negative eigenvector of the so called modularity matrix.
- Walktrap algorithm which is a Repeated Random Walk (RRW) based clustering, *cluster_walktrap* function. Similarly to our MSM algorithm this approach finds modules in a graph by exploiting metastability of the random walk [41], but uses only a time-discrete version of the process.

We compare these methods to our MSM procedure using the largest connected component of our chromatin graph on chromosome 1. As mentioned this comparison is not straightforward since, firstly, none of these methods support fuzzy clustering as in the MSM approach. In particular, the modularity score *Q* which most of these methods use is hard to compare between fuzzy and non-fuzzy clustering and might not be very meaningful in our context. Secondly, the other approaches do not allow us to optimize for MI ratio in an integrated fashion that would impact size and number of modules.

**Fig. 4:**
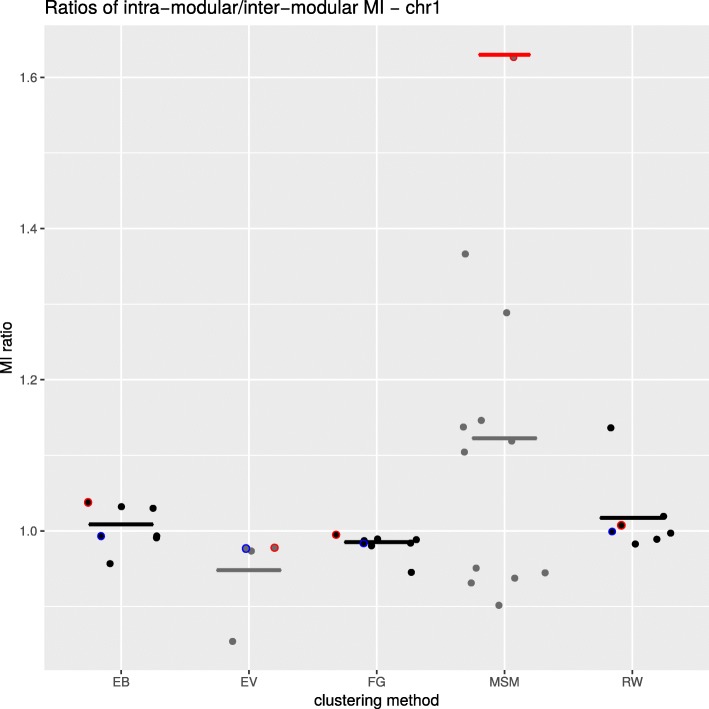
Comparison of different graph clustering methods. Our MSM clustering approach is compared to other methods from the *igraph* package (EB - clustering via edge betweenness; EV-eigenvalue clustering; FG-fast and greedy clustering; RW-random walk clustering). All methods are run with different ranges of parameters and/or number of modules, and the mutual information (MI) ratio is computed for every scenario as described in Material and Methods. For each method the distribution of the resulting MI ratio is shown, together with the median value (horizontal line). For each clustering method the result obtained with the MSM’s optimal number of modules is circled in red and the results obtained with its own optimization is circled in blue. The red line indicates the best partition for our MSM clustering, i.e. values of *α* and *θ* yielding the highest MI ratio

To address these issues, we evaluated a range of different modules for each of the considered methods from the *igraph* package, mimicking optimization for MI ratio. First, we run each algorithm unbiased and assess the modules returned by the optimization algorithm underlying the method. As additional information to this clustering, most of the considered algorithms return a hierarchical overview of the best clusterings for a range of different module numbers - comparable with the variation of the parameters of MSM. This allows us to assess the results for clusterings corresponding to a range of module numbers from 8 to 24 in incremental steps of 4. An exception to this procedure is the EV algorithm that does not offer a simple way to change the number of modules. Rather, we can only influence this number indirectly using the ’steps’ parameter, which can only increase the number of modules until an upper limit is reached. The resulting MI ratios are visualized in Fig. 4. In a second type of assessment, we transfered the information on module number we derived from our MSM approach after optimizing for MI ratio to the other approaches, meaning, we enforced the module number we found with MSM for the other approaches. The outcome of this assessment can also be seen in Fig. 4 marked in red.

Our mehtod returns on average the highest MI ratio compared to other methods (Fig. 4). It is noteworthy that the clustering with the number of modules reported by MSM is often the best clustering and always better or equal to the default clustering.

### Module functional enrichment analysis

GO functional enrichment and pathway analysis from the KEGG database for the genes contained inside each identified module was done with the R package *GSEABase* [42], in order to transfer functional annotation gained from the genes to the lncRNAs contained in the same module. Only enriched terms with adjusted *p*-values lower or equal than 0.1 and having more than two genes from the module annotated with that term are reported in Additional file 2. Nodes not uniquely assigned to a single cluster, but belonging to the transition region defined above, can be also functionally annotated by transferring annotation from their direct neighboring genes.

## Results

In this section we first focus on the analysis of different centrality measures for lncRNA nodes and other annotations, as well as “connectors” lncRNAs of high betweenness. We show that network properties are related to specific regulatory annotations as well as biological functions. Next, we exploit the modularity of the K562 ChIA-PET interaction network to identify network modules including potentially functional lncRNA with fuzzy MSM clustering applied to each chromosome’s biggest component, while still taking into account gene co-expression. Finally, in the absence of an high-throughput gold standard of validated lncRNA functions, we discuss some lncRNA-gene target interactions retrieved manually from the literature and contained in our detected modules, as well as the potential functional importance of inter-modular nodes, which is a unique feature of our approach. We also provide some general means on how to mine the network and the modules to gain a better clue into unknown lncRNA functions.

### Hierarchical graph analysis of the ChIA-PET interaction network

When plotting the frequency of interactions at different genomic distances (Fig. 2, Left panel) one can clearly distinguish two linear ’regimes’, corresponding to a mixture distribution of PETs where two different linear functions can be fitted. The intersection of the two fitted lines in the log-log plot was chosen as cutoff to differentiate self-ligation, corresponding to short range ChIA-PET interactions, from inter-ligation, corresponding to long range interactions. Self-ligation PETs were excluded from the network analysis as, in most of the cases, they do not correspond to chromatin interactions between different genomic segments. Most of the remaining PETs could be annotated as either genes or lncRNAs or other regulatory elements, while about one third of them could not be assigned to any genomic or regulatory annotation (Fig. 2 right panel). In total, 6500 lncRNAs were expressed above the threshold (see “Methods”) in K562 cells, but only 3229 were found to be involved in ChIA-PET interactions. About 40% of the lncRNA-nodes could be annotated with more than one lncRNA (mainly one of the sense and the other on the reverse strand).

To cope with the size and heterogeneous nature of the chromatin graph we developed an hierarchical analysis approach that enabled us to add step-wise resolution to subgraphs of interest guided by the results of the previous step (Fig. 1). First, we analyzed the chromatin graph (Table 1) to identify global hubs by computing the degree centrality of lncRNAs and other genomic elements. An overview of the general properties of the chromatin graph is given in Table 1. The chromatin network is very sparse, with many components representing singleton nodes or containing very few nodes. When looking at the chromatin graph, we notice that only few lncRNAs have a degree centrality higher than 10, while the majority of lncRNAs exhibits a degree between one and three (Additional file 1: Figure S1d). The logarithmic visualization of degrees in Additional file 1: Figure S2 middle panel matches the general observation that in biological networks degrees are often distributed according to a power law, i.e., there exist few hubs and many much less densely connected nodes [22]. A comparison of degree distributions for lncRNAs, protein coding genes, enhancers, promoters/transcribed regions and CTCF sites (Additional file 1: Figure S2) showed that protein-coding genes had the largest degree, constituting the main network’s hubs, followed by lncRNAs (both gene-overlapping and intergenic ones), enhancers, promoters and lastly CTCF sites. Nodes with different annotations followed a power law with similar exponents, except nodes annotated with CTCF sites, probably to reflect the different biological role of such binding sites, as chromatin barriers or insulators [43] with respect to other genomic annotations. For future studies, the top 20 highest-degree lncRNAs from the chromatin network are listed in Table 2.

**Table 1:** Properties of the chromatin graph

chr	no.cc	Min cc csize	Mean cc csize	Max cc csize	Number of nodes containing lncRNA	Nodes containing lncRNA involved in interactions	Node containing lncRNA with highest degree	Degree
chr1	1765	1	3.35	503	444	404	RP11-442N24__B.1,RNU11	26
chr2	1264	1	2.77	64	299	250	ZFP36L2	19
chr3	994	1	2.82	70	213	185	TERC	13
chr4	649	1	2.66	52	160	127	RP11-539L10.3,AC093323.3	9
chr5	890	1	2.71	43	245	197	ARRDC3	15
chr6	1172	1	3.21	337	208	190	ABT1	14
chr7	1016	1	2.76	88	196	164	LINC01287	14
chr8	619	1	2.87	105	187	159	YWHAZ	20
chr9	505	1	3.1	141	127	118	SNHG7	11
chr10	685	1	2.84	92	144	126	VIM	9
chr11	858	1	3.03	128	216	188	BEST1,FTH1	12
chr12	655	1	3.25	69	224	199	BTG1	22
chr13	280	1	2.45	39	62	51	MIR17HG	21
chr14	357	1	2.98	36	131	115	PRMT5	10
chr15	430	1	3	72	156	132	GABPB1	9
chr16	457	1	3.63	196	182	166	RAB26,TRAF7	17
chr17	517	1	4.3	350	275	267	LINC00910	17
chr18	278	1	2.61	71	61	47	MYL12A	17
chr19	380	1	5.36	158	227	220	SLC1A5	15
chr20	407	1	3.18	90	109	99	CEBPB	26
chr21	207	1	3.02	53	73	62	DYRK1A	7
chr22	279	1	3.24	67	84	78	POLDIP3	20
chrX	358	1	2.75	60	57	49	VSIG4	10

For each chromosome we report: the total number of connected components (*no.cc*), the minimum number of nodes (*min cc csize*), the average number of nodes (*mean cc csize*) and maximum number of nodes (*max cc csize*)) of the connected components, the total number of annotated lncRNAs (*number of lncRNAs*), the total number of lncRNAs which are involved in at least one interaction (*lncRNAs in interactions*), the lncRNA gene symbol of the highest degree’s lncRNAs (*lncRNA with highest degree*) and the actual highest degree value for that lncRNA (*degree*)

**Table 2:** Top 20 lncRNAs with highest degree from the chromatin graph

lncRNA name	Degree	To-gene degree	Chormosome	Annotation	RPKM	Conserved	Disease
RP11-442N24__B.1,RNU11	26	9	chr1	lnc_transcr_enhancer	227.4193	no	yes
RP4-798A10.7	21	5	chr1	lnc_transcr_enhancer	16.0232	no	no
MIR17HG	21	1	chr13	lnc_transcr_enhancer	518.9101	no	yes
LINC00910	17	5	chr17	lnc_transcr_enhancer	37.0585	no	no
RP11-1082L8.4	16	2	chr8	lnc_transcr_enhancer	1.2984	no	no
LINC01287	14	0	chr7	lnc_transcr_enhancer	77.8624	no	no
TERC	13	3	chr3	lnc_CTCF	111.0789	no	yes
AC073283.4	13	3	chr2	lnc_transcr_enhancer	0.156	no	no
RP11-495P10.3	12	0	chr1	lnc_promoterFl	1.0816	no	no
RP11-301G19.1	12	0	chr6	lnc_transcr_enhancer	914.2072	no	no
KB-1732A1.1	12	1	chr8	lnc_transcr_enhancer	1.0105	no	no
SNHG7	11	5	chr9	lnc_TSS	112.0253	no	no
BZRAP1-AS1	11	0	chr17	lnc_transcr_enhancer	6.9533	no	no
RP11-247A12.2	10	3	chr9	lnc_transcr_enhancer	0.3745	no	no
CTD-2587H24.5	10	8	chr19	lnc_transcr_enhancer	0.1669	no	no
CTD-2587H24.10	10	7	chr19	lnc_transcr_enhancer	17.1446	no	no
SNHG16,RP11-666A8.8	9	4	chr17	lnc_promoterFl	73.5648	no	yes
SNHG12	9	7	chr1	lnc_TSS	229.3524	no	no
RP5-884M6.1	9	2	chr7	lnc_transcr_enhancer	30.2113	no	no
RP11-539L10.3,AC093323.3	9	6	chr4	lnc_transcr_enhancer	92.453	no	no

For each lncRNA we report its degree centrality (*degree*), its degree centrality computed only from gene connections (*to-gene degree*), the chromosome it belongs to (*chromosome*), its annotation based on chromatin segmentation (*annotation*), its expression value (RPKM) in the K562 cell line (*expression*), whether it is positionally conserved according to X et al. [31] (*conserved*), and whether it is known from databases or literature its involvement in diseases(*disease*)

Since the chromatin graph decomposes in a natural way into the graphs representing the single chromosomes, we compute the lncRNA degree chromosome-wise. Even nodes that are not among those of highest degree in the chromatin graph may be distinguished with respect to their chromosome graph. Second, we focus on the connected components containing lncRNAs of each chromosome graph to obtain the next resolution level. Small components are then amenable to a full analysis of different aspects of interest, while for large connected components we still need indicators that guide our search for important lncRNA modules. In (Additional file 1: Tables S2, S3 and S4) we report this analysis for the biggest connected components of chromosome 1, 17 and 11, respectively. In addition, we evaluate the betweenness centrality of each lncRNA node. Among lncRNAs with high betweenness in their respective connected component we find MALAT1, SHG16, RNU11 and RP11-400F19.8, known oncogenes, as well as lncRNAs of unknown function, such as LINC00910, RP11-442N24 and RP4-798A10.7. Interestingly, PETs annotated as lncRNAs, which overlapped also a protein coding gene, either on the same or the anti-sense strand, had on average the highest betweeness compared to other genomic classes, including protein coding genes (Additional file 1: Figure S2 right panel, Table S1). This points to the important central role of these regions with dual genomic annotation (coding/non-coding) as linkers and communicators between different regulatory modules in the ChIA-PET network. Finally, to identify relevant functional units we conduct a module search using the MSM clustering method described above.

### Network analysis and biological properties of lncRNAs

By manually inspecting the functional annotation of the top 20 expressed lncRNAs with highest degree, we find several lncRNAs known from previous studies to be cancer-associated. For example, RNAs from the SNHG family important in cell proliferation and invasion in different cancer types [44]; RP11-301G19.1, over-expressed in leukemia [45]; TERC, involved in telomerase activity and associated to leukemic cells [46], and the intergenic lncRNA MIR17HG, host transcript of the MIR-17-92a-1 cluster, known to be involved in cell survival and cancer proliferation [47]. However, disease annotation is sparse and limited for lncRNAs compared to protein-coding genes. The fraction of intergenic long non-coding RNAs (lincRNAs) from the ChIA-PET network, that could be annotated with a disease in our analysis (see “Methods” section for more details) was only 9% (217 out of 2305), therefore it is hard to systematically access whether high-degree lncRNAs are significantly associated to diseases. Comparing the degree distribution of lincRNAs annotated with a disease versus lincRNAs not linked to a disease we do not observe any significant associations (*p*-value =0.384, Wilcoxon rank sum test). When we perform the same analysis including also lncRNAs overlapping protein-coding genes, we can assign a disease up to 42% of the lncRNAs in our network, and obtain a significant association between degree centrality and disease annotation (*p*-value <1.22∗10^−16^, Wilcoxon rank sum test, Additional file 1: Figure S3).

A recent study from Liu et al. [33] investigates the functional importance of lncRNAs, mainly as *trans* regulators of gene expression, by performing CRISPR interference and targeting thousands of lncRNA loci in seven diverse cell lines, including K562. We partly used these data to explore other biological properties of our ChIA-PET network. Liu et al. define functional lncRNAs or ’hits’ those which showed a significant phenotype, i.e. affecting cell growth, in a cell-type specific manner. K562 hits were enriched in the chromatin graph, compared to non-hits (odd ratio = 2.07, p=0.008, Fisher’s exact test), but did not have significantly higher degree centrality. K562 lncRNAs annotated by Liu et al. to be in close genomic proximity to cancer risk SNPs were also enriched in the chromatin network compared to lncRNAs far from those SNPs (odd ratio = 2.65, p=1.2* 10−5, Fisher’s exact test) but did not have significantly higher degree.

LncRNAs annotated as enhancers from *chromHMM* were enriched for tissue-specific expression (odd ratio=2.4, *p*-value=1.4*10-7, Fisher’s exact test) and had significantly higher degree centrality compared to lncRNAs which did not overlap enhancer elements (*p*-value= 5.4∗10^−62^, Wilcoxon rank sum test, Additional file 1: Figure S3). This still holds when considering also lncRNAs overlapping protein coding genes (*p*-value= 1.3∗10^−48^, Wilcoxon rank sum test, Additional file 1: Figure S3). The significant associations between high degree and enhancer annotation of lncRNAs in the network holded also for FANTOM5 enhancers (*p*-value=0.034, Wilcoxon rank sum test) and enhancers defined by other studies (*p*-value= 2.1∗10^−9^, Wilcoxon rank sum test), as well as super-enhancers (*p*-value= 4.5∗10^−10^, Wilcoxon rank sum test) [32]. This suggests that enhancer-like lncRNAs are hubs in the Pol II-mediated ChIA-PET network and connect several regulatory regions to gene loci in an extensive and combinatorial fashion. In addition, lncRNAs which overlapped both ’enhancer’ and ’transcribed’ annotations from chromHMM, which we denote as ’transcribed enhancers’, had a significantly higher degree compared to those lncRNAs annotated only as ’enhancer’ but not ’transcribed’ from chromHMM (*p*-value= 1.9∗10^−19^, Wilcoxon rank sum test). All these findings are in line with the results from Liu et al., where the authors show that enhancer proximity and chromosomal contacts correlate with lncRNA function [33], and that the transcription of the lncRNA itself might confer, in some cases, the regulatory potential to the lncRNA genomic locus.

Positionally conserved lncRNAs from our network, defined as lncRNAs located close to genes which are orthologous between human and mouse [31], had also a significantly higher degree compared to the non positionally conserved ones (*p*-value=0.044, Wilcoxon rank sum test, Additional file 1: Figure S3). Finally, also lncRNAs which had a direct orhtologous gene in mouse [33] had significantly higher degree than the rest (*p*-value=0.037, Wilcoxon rank sum test), highlighting the importance of evolutionary conserved lncRNAs in the ChIA-PET chromatin network. Positionally-conserved lncRNAs have also been associated with developmental or cancer genes, and shown to be in chromatin loops, which contact enhancer-regulatory sequence. In our network, we observe that lncRNAs which are also annotated as positionally conserved, have a significantly higher degree than not positionally conserved ones (*p*-value = 0.044, Wilcoxon rank sum test), indicating their potential role as functional hubs in the Pol II chromatin network.

### Small network components contain validated lncRNA-gene functional interactions

Prior to cluster analysis, we first inspected small connected components of the network (sub-graphs of the order of tens of nodes) to assess whether the spatial proximity in the Pol II ChIA-PET network recapitulates some well known lncRNA-gene target interactions from the literature. The lncRNA transcript overlapping the longest intron of the AHI1 gene has been shown to significantly impact the expression of the BCLAF1 in K562 from a CRISPRi experiment [33]. Here we show that this regulation is mediated by a direct ChIA-PET interaction in a small connected network module on chromosome 6 (Additional file 1: Figure S4a)). In addition, other ChIA-PET modules contain validated interactions of lncRNAs with their target genes, for example PVT1 with its known target MYC [33] on a small connected component on chromosome 8 (Additional file 1: Figure S4b)). A small module on chromosome 1 contains the lncRNA CYP4A22-AS1 (Additional file 1: Figure S4d)), also known as ncRNA-a3, which has been shown to act as enhancer for its flanking stem cell leukemia-associated gene TAL1 [13], and we recapitulate the direct interaction between them. The active enhancer-like lncRNA linc00853, also known as ncRNA-a4 is also part of the ncRNA-a3 network (Additional file 1: Figure S4d)) and directly regulates its flanking gene CMPK1, as already previously verified experimentally [13], suggesting a synergistic action of these two lncRNAs in coordinating the transcriptional activity of a group of four genes in this module.

Finally, we looked at the well characterized lncRNA Xist (Additional file 1: Figure S4e)), known to be involved in transcriptional gene silencing during X-chromosome inactivation. From our analysis it is evident, given the lack of ChIA-PET interactions, that Xist does not associate to Pol II to regulate its target genes in an enhancer-like fashion, as expected given its suggested silencing function. On the other hand, we could recover direct Pol II-mediated interactions between XIST and lncRNA FTX, JPX and TSIX (Additional file 1: Figure S4e) which are known regulators of XIST transcription [11].

### Analysis of lncRNA-containing modules

For the biggest components of each chromosome we performed MSM clustering as described in Material and Methods and inspected the resulting modules for functional annotation. One way to gain functional clues about uncharacterized lncRNAs is to inspect the functions of its interacting genes or the genes contained in the same module via GO/KEGG term enrichment. Many of the identified clusters in our network were enriched in cancer-related terms (Additional file 2). The most abundant KEGG pathway from our analysis “Chronic myeloid leukemia” is found in ten modules, in line with K562 being a leukemia cell line, though only two modules in chromosome 17 contained more than one gene in the pathway. The more general term “Pathways in cancer” was enriched in four modules (see Additional file 2, sheet 2). We found more enriched terms linked to cancer, such as “mTOR signaling pathway”, “Jak-STAT signaling pathway”, “hematopoietic stem cell differentiation” and “response to interleukin-15” (hematopoietic growth factor (Additional file 2).

#### Examples of modular structures and putative lncRNA functions on chromosomes 20, 1, 17 and 11

**Fig. 5:**
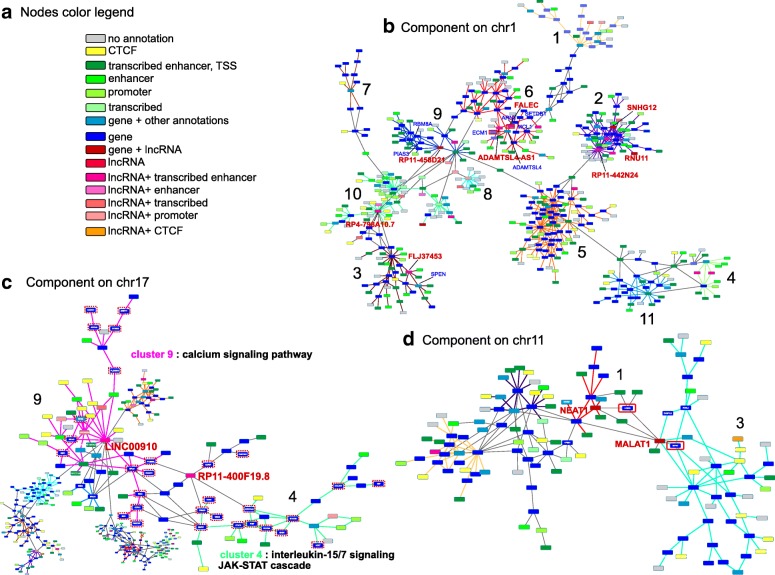
Module detection. **a** Node color legend, **b** chromosome 1 biggest component, **c** chromosome 17 biggest component and **d** chromosome 11 biggest component. Nodes are colored according to their overlaid genomic annotation and/or chromatin states (**a**). Relevant lncRNAs and connected genes are highlighted as text. Known and validate cancer genes in **c**) are circled in red with dotted lines. Different modules from the clustering analysis are represented via different edges’ colors. ’Fuzzy’ nodes, i.e. nodes which could not be confidently assigned to any module, have connections colored in gray departing from them

We detect two modules on the biggest component of chromosome 20, where cluster 1 contains the validated functional interaction between TRERNA1 lncRNA and the SNA1 gene [8], mediated by several enhancer elements (Additional file 1: Figure S4c). We detect 11 modules for the biggest component on chromosome 1 obtained with setting *α*=1000 and *θ*=0.8 (Fig. 5b). Different clusters are recognizable by different colors of their intra-cluster edges. Cluster 6 in Fig. 5b is the regulatory module containing the well-known eRNA FALEC, which has been shown to harbor enhancer-like functions and significantly influence the expression of its flanking gene ECM1 [13], also present in the same module. By inspecting the interactions in this module, we learn that the interaction between FALEC and the ECM1 locus is mediated by the ADAMTSL gene. Interestingly, the Myeloid Cell Leukemia apoptosis regulator MCL1 and the Hypoxia-Inducible factor 1-Beta ARNT are also in the same module, indirectly linked to FALEC via other lncRNAs, protein-coding genes and several transcribed enhancer elements (colored in green in Fig. 5b). The module containing FALEC is enriched in functional terms related to chromatin silencing and negative regulation of gene expression, highlighting a role of this lncRNA in epigenetic-related processes. Other interesting modular units on chromosome 1 comprise: cluster 3, containing the lncRNA of unknown function FLJ37453 which is connected via intra-modular interactions to SPEN, known to associate to lncRNAs (e.g. Xist) and mediate gene repression [48]; cluster 2, containing, among others, lncRNAs SNHG12, ubiquitously expressed in several cancers and two other lncRNAs of high degree, RUN11 and RP11-442N24, as well as genes interestingly enriched in functions associated with lipid metabolism, transcription termination and p53-mediated signal transduction; cluster 10, containing the high degree lncRNA RP4-798A10.7 and genes with enriched functions related to chromatin and nucleosome assembly, suggesting a role of this lncRNA in shaping chromatin organization.

Clustering of the biggest component on chromosome 11 (Fig. 5d) resulted in an optimal partition of four dominant modules, obtained with setting *α*=1000 and *θ*=0.7 (thus identifying more relaxed modules). Particularly interesting are the modules in Fig. 5d) marked by red and light blue connections, namely cluster 1 and 3, linked by the oncogene lncRNA MALAT1, known to act as transcriptional regulator for numerous genes involved in cancer metastasis and cell migration [9]. MALAT1 has a degree of 8, but exhibits a very high betweenness. This indicates that MALAT1 is important in the context of the entire connected components, not only for its first-order neighbors, but also because it brings different gene clusters in close proximity at the chromatin level. This fits well with the known role of MALAT1 as global regulator of cancer genes and orchestrator of a global transcriptional response [9]. While the physical interaction of lncRNA MALAT1 with the SIPA1 leukemia oncogene has been experimentally validated [49] and recapitulated in our network, MALAT1 is linked to other crucial oncogenes in the two linked modules, and co-expressed with NEAT1, another well-known lncRNA in the context of cancer [9].

Finally, we briefly discuss the clustering results of the biggest connected component of chromosome 17, obtained with *α*=700 and *θ*=0.7 (Fig. 5c), resulting in eleven modules. This component is particularly interesting because it contains several lncRNAs with very high degree/betweenness, so potential core players in the leukemia regulatory network, but of unknown function, such as LINC00854 and LINC00910. LINC00910 was already pointed in other studies as a highly connected lncRNA [50], observed to be linked to an upstream super-enhancer [50, 51] and hypothesized to be involved in immune related functions and lymphocyte activation [50]. In our network, it exhibits the highest degree in chromosome 17 and very high betweenness, it interacts with several transcribed enhancers and with many CTCF binding sites. It is also found in direct or indirect interactions with numerous known cancer genes, such as NBR1, BRACA1, ICT1, SUMO, NUP85 and others. As the genes contained in this detected module are enriched in the ’calcium signaling pathway’ annotation, which is a key regulator of B lymphocyte fate in Leukemia [52] we propose a potential function for LINC00910 in the Leukemia’s ChIA-PET as regulator of calcium signaling-related genes. However, experimental approaches so far could not identify genes which are significantly regulated by LINC00910 in the K562 cell line, neither in *cis* or in *trans* [33], and further experimental tools are needed to validate this hypothesis.

Of great interest in this component is lncRNA RP11-400F19.8, a node with high degree and high betweenness, which was not assigned to any cluster by our method but belonged to the previously defined ’transition’ region (Additional file 1: Table S5). Although not assigned to any module, RP11-400F19.8 is far from being a non-functional lncRNA, and was already identified in a previous transcriptome-wide association study as a cancer risk locus [53]. In our network RP11-400F19 links two modules enriched in different immune biological processes (Fig. 5c) and brings in close spatial proximity known cancer genes (denoted with dotted red circles in Fig. 5c)) from both modules.

## Discussion

LncRNAs play key regulatory roles in a wide range of processes, and a small number of them has been shown to operate in the nucleus and influence transcriptional regulation of neighboring or distal genes. To which extent cell-type specific 3D chromatin organization and other DNA regulatory elements contribute to lncRNA-mediated gene regulation has been poorly investigated. In addition, functional annotation for most of the annotated lncRNAs, as well as their role in gene regulatory networks remains elusive. Based on the fact that transcripts sharing common expression patterns should largely share similar biological pathways, a number of different studies have used the ’guilt by association’ approach to functionally annotate lncRNAs based on expression similarities with protein-coding genes of known function.

Here we comprehensively map ChIA-PET chromatin contacts mediated by Pol II in the K562 cell line to lncRNAs, genes and other DNA regulatory elements, and propose a multi-step approach to analyze lncRNA regulatory functions using graph analysis techniques. We first show a significant association between some network properties of lncRNA-annotated nodes, such as degree or betweenness, and biological properties, such as enhancer functions of lncRNAs from multiple datasets. Afterwards, we inspect functional mechanisms of network modules. The added value of our approach is twofold. First, lncRNA-mediated transcriptional regulatory modules are identified by means of fuzzy clustering analysis directly on the chromatin network, providing a first link between transcriptional regulation and lncRNA association/functions at chromatin level. Second, although alternative choices exist for the module search, we decided on the MSM clustering since it does not impose an often artificial full partition of the network into modules, but outputs a fuzzy clustering which allows more flexible interpretation of lncRNA regulation. Also, the *θ* and *α* parameters of our clustering method are chosen to maximize gene co-expression inside the same module, allowing to incorporate additional information in the module refinement, beyond network topology. Our approach can identify direct lncRNA targets, as well as the regulatory modules they belong to, as shown here for known and novel lncRNAs. Although it is not often possible to discern modules of co-regulated genes/lncRNAs from causal lncRNA-mediated *cis*-regulation without experimental validation, and many lncRNAs identified in the modules might actually function in *trans* to regulate gene expression, our approach is promising in detecting *cis*-regulatory modules. In fact, a previous study investigating functional interactions of MALAT1 and NEAT1 with target genes via Capture Hybridization Analysis of RNA targets (CHART) sequencing in the MCF7 cell line shows that half of the interacting genes are located up to three hops of connectivity with their lncRNAs and in the same inter-connected chromatin cluster [50]. Unfortunately the same CHART data are not available for K562, but these observations, together with the evidence of validated direct and indirect lncRNA-gene target functional interactions in the clusters of our network, highlights that the detected modules are a good starting point to look for potential lncRNA-mediated *cis*-regulatory interactions. Our approach, presented here for the Pol II ChIA-PET network in K562, is generally straightforward enough to be applied to other factors, such as CTCF and other cell lines, and module analysis repeated for other connected components.

Incorporation in the future of other annotations in the network, such as Transcription Factor Binding Sites, will enable a better interpretation of individual modules.

Although the modular lncRNA regulatory code remains to be tested, investigating the connections between lncRNAs, genes and other regulatory elements are important steps towards further definition of lncRNA functions on a system-wide level. The investigation of modules related to lncRNAs whose functionality is not yet known can suggest new targets and the regulatory components involved in regulation. Therefore, we propose that our functional annotation scheme can be applied to thousands of lncRNAs in a tissue-specifc manner.

## Conclusion

In this study we demonstrate that the integration of 3D chromatin interaction and co-expression analysis provides a powerful network analysis approach for in silico functional analysis of both known and novel lncRNAs involved in transcriptional regulation. The results presented here, in particular the detected regulatory modules on the ChIA-PET interaction network, are an important resource for further biological research.

## Additional files

Additional file 1 This pdf includes supplementary tables and figures referred to in the main text. This includes four additional figures, included one showing several small connected components pointing to lncRNA whose regulated genes are known from literature, as well as tables with network properties of lncRNAs in the biggest connected components of chromosomes 1, 11 and 17. (PDF 421 kb)

Additional file 2 In this excel file we provide the results from clustering analysis of the biggest connected component of each chromosome, in order to assist future experimental studies. For each component, we report the results from those values of *α* and *θ* yielding the best partition according to the MI ratio criteria. We report the clustering parameters, the resulting MI ratio, the number of obtained modules per component, the number of lncRNAs, protein-coding gene, as well as the overall number of nodes for each component. Note when the genomic coordinates of a gene and a lncRNA overlap, both the gene and the lncRNA name are reported for the same node. Sheet 2 of this file contains the results of GO/KEGG enrichment analysis for the modules of each chromosome’s biggest component. (XLSX 25 kb)

## Abbreviations

CHART: Capture hybridization analysis of RNA targets
ChIA-PET: Chromatin interaction analysis by paired-end tag sequencing
CSHL: Cold spring harbour lab
EB: Edge betweeness
EV: Eigenvalue
FG: Fast greedy
GO: Gene ontology
KEGG: Kyoto enciclopedia of genes and genomes
lincRNAs: Intergenic long non-coding RNAs
lncRNAs: Long non-coding RNAs
MI: Mutual information
MSM: Markov state model
ncRNAs: Non-coding RNAs
PETs: Paired-End tags
Pol II: Polymerase II
RBPs: RNA binding proteins
RNA-seq: RNA sequencing
RPKM: Reads per kilobase of transcript per million mapped reads
RRW: Repeated random walk
SNP: Single nucleotide polymorphism
TSS: Transcription start site
