# Supplementary material for: Identifying lncRNA-mediated regulatory modules via ChIA-PET network analysis
Source: BMC Bioinformatics. 2019 May 29;20:292. doi: 10.1186/s12859-019-2900-8 (PMC6540383; doi:10.1186/s12859-019-2900-8)
Supplement: Supplementary file 1 — This pdf includes supplementary tables and figures referred to in the main text. This includes four additional figures, included one showing several small connected components pointing to lncRNA whose regulated genes are known from literature, as well as tables with network properties of lncRNAs in the biggest connected components of chromosomes 1, 11 and 17. (PDF 421 kb) [file 12859_2019_2900_MOESM1_ESM.pdf]

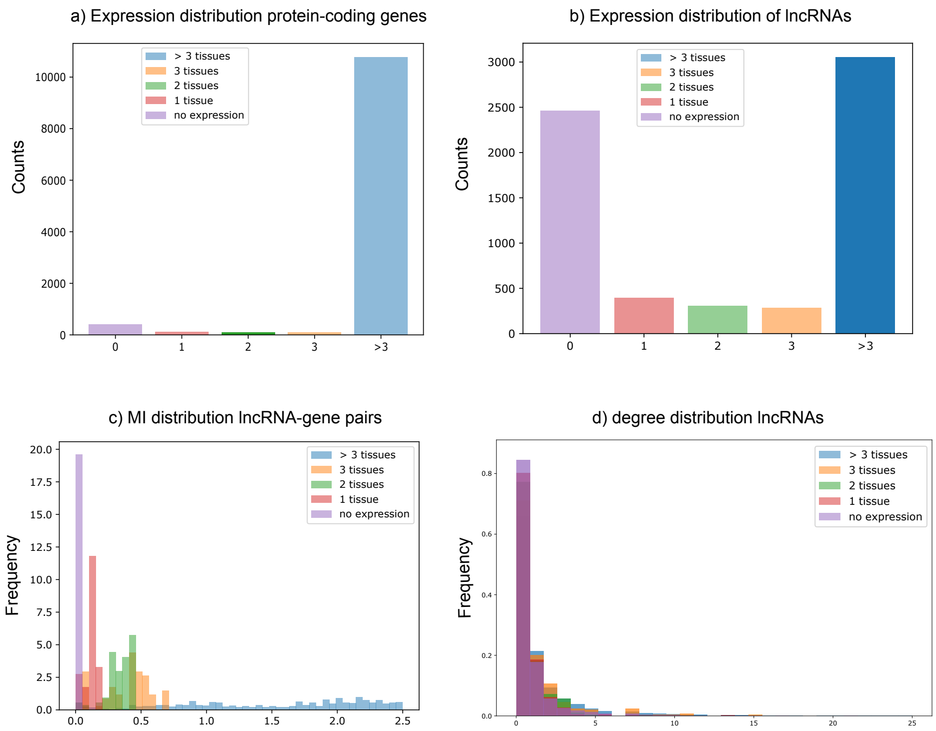

Figure S1: **Cell line specificity analysis of the network nodes.** a) Distribution of expression of protein coding genes for tissue-specific and ubiquitously expressed gene determined from 24 ENCODE cell lines. b) Distribution of expression of lncRNAs for tissue-specific and ubiquitously expressed lncRNAs determined from 24 ENCODE cell lines. 'No expression' refers to lncRNA with no detectable expression in any of the ENCODE cell lines (whole cell RNA-seq) but with detectable expression in the RNA-seq libraries of the chromatin RNA fraction in K562, reflecting expression of nascent transcripts. c) Distribution of gene-lncRNAs Mutual Information values (MI) for tissue-specific and ubiquitously expressed lncRNAs computed from 24 ENCODE cell lines. d) Degree distribution for tissue-specific and ubiquitously expressed lncRNAs.

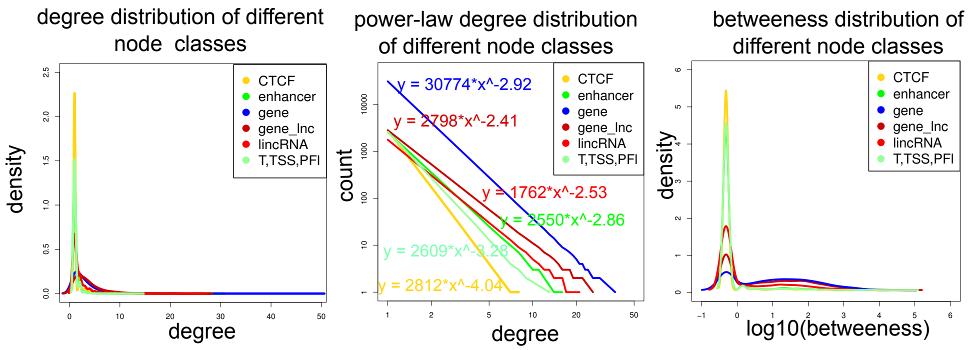

Figure S2: **Network properties of different annotation classes** Left panel: degree distribution of different nodes' annotations from the chromatin network; middle panel: power law degree distribution of different nodes' annotations; right panel: log betweenness distribution of different nodes' annotations. A pseudocount of 0.5 has been added to nodes with a betweeness value of zero.

Table S1: Network properties of different genomic regions

annotation	min_d	q1_d	median_d	mean_d	q3_d	max_d	min_b	q1_b	median_b	mean_b	q3_b	max_b
CTCF	1	1	1	1.09	1	8	0	0	0	3.34	0	1410
enhancer	1	1	1	1.18	1	30	0	0	0	31.5	0	61845
gene	1	1	2	2.78	4	49	0	0	3	211.67	51	62024.27
gene_lnc	1	2	3	3.51	4	26	0	0	12	236.41	85.375	27307.88
lincRNA	1	1	1	1.87	2	26	0	0	0	134.99	4.895	45314.76
T,TSS,PFI	1	1	1	1.14	1	14	0	0	0	30.52	0	84898.53
unknown	1	1	1	1	1	1	0	0	0	0	0	0

For each genomic annotation we report minimum degree, 25% lower quantile of the degree distribution, median degree, mean degree, 75% upper quantile of the degree distribution and maximum degree. The same quantities are reported for different annotations regarding the betweenness distribution.

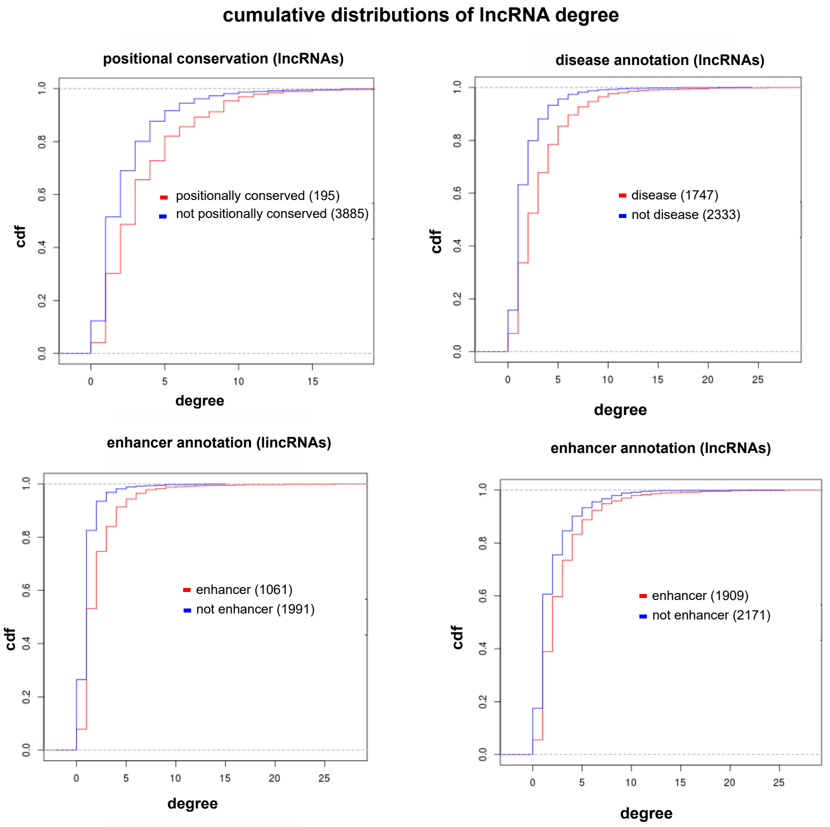

Figure S3: **Cumulative distributions of lncRNA node degree for different biological properties.** Upper left: positional conservation for lncRNAs; upper right: disease annotation for lncRNAs; lower left: chromHMM enhancer annotation for lincRNAs; lower right: chromHMM enhancer annotation for lncRNAs. Numbers in brackets indicate the sample size, i.e. the number of lncRNAs corresponding to each cumulative distribution.

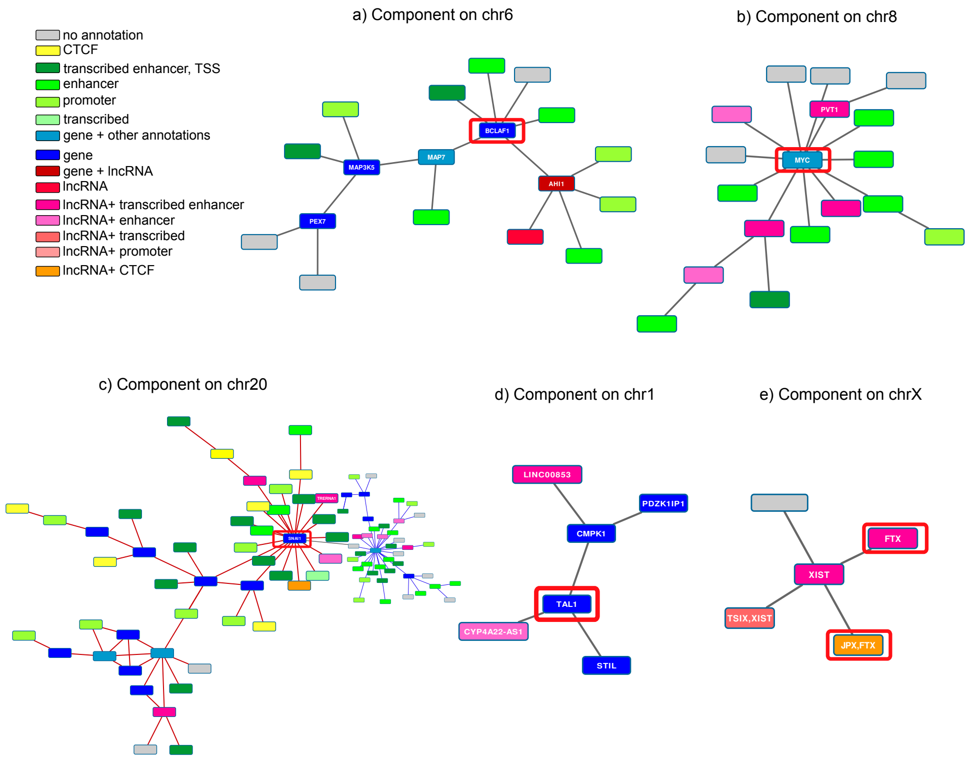

Figure S4: **Analysis of small connected components** a) Small connected component on chromosome 6 highlighting the functionally validated interactions between the lncRNA overlapping the AHI1 gene and BCLAF1 (circled in red). b) Small connected component on chromosome 8 highlighting the validated regulatory interaction between PVT1 lncRNA and MYC (circled in red). c) Connected component on chromosome 20 highlighting the validated regulatory interaction between TRERNAI lncRNAs and SNAI1 gene (circled in red). d) Small connected component on chromosome 1 highlighting two validated regulatory interactions of the two enhancer-like lncRNAs ncRNA-a3 (CYP4A22-AS1) and ncRNA-a4 (linc00853) on TAL1 gene (circled in red). e) Small connected component on chromosome X involving the lncRNA Xist, major effector of the X inactivation process. Validated regulators of Xist (FTX and JPX) are circled in red.

Table S2: Network properties of lincRNAs in the biggest connected component of chromosome 1

lncRNA	degree	to_gene degree	betweenness	expression
RP11-442N24__B.1,RNU11	26	12	28958.62	5.465
RP4-798A10.7	21	6	32092.38	16.0232
RP11-495P10.3	12	0	5456	1.0816
SNHG12	9	7	2514.22	229.3524
RP11-458D21.1	9	6	22924.47	52.9613
ADAMTSL4-AS1	6	3	2249	50.9546
FLJ37453	5	3	4126.5	7.1451
RP1-212P9.3	4	1	397.41	0.5435
RP11-337C18.8,RP11-337C18.10	3	1	250.67	0.044
CH17-373J23.1	3	0	6056.3	35.8141
SEC22B	2	1	0	26.7178
RP5-888M10.2	2	2	0	1.2264
RP5-1092A3.5	2	1	0	2.3792
RP4-798A10.2	2	1	501	14.2565
RP1-37C10.3	2	2	0	0.1157
RP11-474O21.5	2	2	0	111.6587
RP11-337C18.10	2	0	249.33	0.0371

For each intergenic lncRNA we report its name (*lncRNA*), its degree centrality inside the connected component (*degree*), its degree centrality computed only from gene connections (*to-gene degree*), its betweenness (*betweenness*) and its expression value (RPKM) in the K562 cell line (*expression*).

Table S3: Network properties of lincRNAs in the biggest connected component of chromosome 17

lncRNA	degree	to_gene degree	betweenness	expression
LINC00910	17	8	45314.76	37.0585
SNHG16,RP11-666A8.8	9	5	8527.74	73.5648
LINC00854	8	3	1776.27	70.9481
RP13-516M14.1,RP13-516M14.4	6	2	1834.12	62.6496
RP11-1055B8.9	6	4	513.68	9.3433
RP11-400F19.8	5	5	7618.89	3.1249
RP13-766D20.4	4	3	1130.2	1.2599
LINC00482	4	2	348	15.868
CTD-2020K17.1	4	4	423.66	8.9101
RP11-285E9.6	3	2	1040	0.0321
RP11-1055B8.3	3	1	695	0.5555
RP11-1055B8.10,RP11-1055B8.4	3	1	695	3.095
CTD-3014M21.1	3	0	35.83	6.4664
RP11-666A8.8	2	0	0	2.8546
RP11-392O1.4	2	1	10.47	0.0438
RP11-358B23.1	2	1	173.5	0.9049
RP11-1055B8.3,RP11-1055B8.10,RP11-1055B8.4	2	1	348	0.5555
MAP3K14-AS1	2	1	348	12.5812
CTD-2561B21.11	2	1	348	8.7781

For each lincRNA we report its name (*lncRNA*), its degree centrality inside the connected component (*degree*), its degree centrality computed only from gene connections (*to-gene degree*), its betweenness (*betweenness*) and its expression value (RPKM) in the K562 cell line (*expression*).

Table S4: Network properties of lincRNAs in the biggest connected component of chromosome 11

lncRNA	degree	to_gene degree	betweenness	rpkm
MALAT1	8	6	4023.77	10426.3926
NEAT1	6	4	361.67	1685.197
MIR194-2HG	2	2	0	3.3122

For each lincRNA we report its name (*lncRNA*), its degree centrality inside the connected component (*degree*), its degree centrality computed only from gene connections (*to-gene degree*), its betweenness (*betweenness*) and its expression value (RPKM) in the K562 cell line (*expression*).

Table S5: Fuzzy clustering or inter-modular nodes

lncRNA	chr	type	expr	degree
RP11-661A12.5	chr8	lnc_TSS	11.36	2
RP11-838N2.5	chr18	lnc_enhancer	0.65	6
AC073842.19	chr7	lnc_transcr_enhancer	1.06	13
RP11-400F19.8	chr17	lnc_transcr_enhancer	3.13	5
RP13-516M14.1	chr17	lnc_transcr_enhancer	62.65	6
RP11-388C12.5	chr17	lnc_TSS	9.55	1
RP4-798A10.2	chr1	lnc_transcribed	14.26	1
CH17-373J23.1	chr1	lnc_promoter	35.81	3
RP11-403I13.5	chr1	lnc_enhancer	0.27	1
CTD-2555K7.2	chr14	lnc_transcr_enhancer	1.1	2
RP11-298I3.4	chr14	lnc_CTCF	0.74	3
RP11-57206.1	chr3	lnc_transcribed	35.16	2
U91328.21	chr6	lnc_TSS	0.15	2

For each lncRNA we report its name (*lncRNA*), the chromosomal location (*chr*), its genomic annotation (*type*), its expression value (RPKM) in the K562 cell line (*expr*) and degree centrality (*degree*)
